# Gnathodiaphyseal dysplasia with a novel genetic variant in a large family from Iran

**DOI:** 10.1002/mgg3.2004

**Published:** 2022-06-27

**Authors:** Vahid Reza Yassaee, Arash Khojasteh, Farzad Hashemi‐Gorji, Hossein Sadeghi, Hannaneh Safiaghdam, Reza Mirfakhraie

**Affiliations:** ^1^ Genomic Research Center Shahid Beheshti University of Medical Sciences Tehran Iran; ^2^ Department of Medical Genetics, School of Medicine Shahid Beheshti University of Medical Sciences Tehran Iran; ^3^ Dental Research Center, Research Institute of Dental Sciences Shahid Beheshti University of Medical Sciences Tehran Iran; ^4^ Student Research Committee, Dental School Shahid Beheshti University of Medical Sciences Tehran Iran

**Keywords:** *ANO5*, bone dysplasia, bone fractures, exome sequencing, gnathodiaphyseal dysplasia (GDD)

## Abstract

**Background:**

Gnathodiaphyseal dysplasia (GDD) is an ultrarare autosomal dominant bone dysplasia characterized by cementoosseous lesions of the jawbones, bone fragility, frequent bone fractures at the young age, bowing of tubular bones, and diaphyseal sclerosis of long bones associated with generalized osteopenia. GDD is caused by point mutations in *anoctamin‐5* (*ANO5*) on chromosome 11p14.3. For the past few years, next generation sequencing (NGS) technology has facilitated the discovery of causative variants in genetically heterogeneous diseases.

**Methods:**

In this study, exome sequencing (ES) was performed using the DNA sample of the proband. Family histories and clinical information were collected through comprehensive medical examination and genetic counseling.

**Results:**

ES results identified a heterozygous variant, NM_213599.3:c.1078T>C(p.Cys360Arg) in the *ANO5* gene. Sanger sequencing was performed to confirm the detected pathogenic variant in DNA samples of the entire family (except deceased individuals), which segregated with the disease within the family. Finally, in silico analysis was applied to test the pathogenicity of the variant using various online software.

**Conclusion:**

In summary, our investigation identified a novel pathogenic variant in the *ANO5,* responsible for gnathodiaphyseal dysplasia in a large Iranian family. Therefore, based on the present study, this variant can be helpful for diagnosis and effective management of GDD patients.

## Abstract 2

Gnathodiaphyseal dysplasia (GDD) is an ultrarare autosomal dominant bone dysplasia characterized by cementoosseous lesions of the jawbones, bone fragility, frequent bone fractures at the young age. In this study, Exome Sequencing results identified a heterozygous mutation, NM_213599.3:c.1078T>C(p.Cys360Arg) in the ANO5 confirmed by Sanger sequencing.

## 1 INTRODUCTION

Gnathodiaphyseal dysplasia (GDD; OMIM#166260) is an ultrarare autosomal dominant generalized skeletal syndrome characterized by fibro‐osseous lesions of jawbones and sclerosis of tubular bones (Andreeva et al., 2016). In this skeletal syndrome, a phenotype of bone fragility as well as lesions in the mandible can be observed, which can result in facial deformities and susceptibility to purulent osteomyelitis (Ahluwalia et al., 2007). The fragility is attributed to diaphyseal sclerosis and cortical thickening of tubular bones, while most lesions of the mandible resemble those of florid osseous dysplasia (Riminucci et al., 2001).

GDD can be confused with other syndromes such as fibrous dysplasia (FD) or McCune‐Albright syndrome (MAS) as it shares most of their clinical and histopathological features (Nishimura et al., 1996; Vengoechea & Carpenter, 2015). Indeed, it has been previously described as gigantiform cementoma (GC) or osteogenesis imperfect (OI; Akasaka et al., 1969; Moshref et al., 2008). The GDD like OI patients have shown recurrent fractures, but in contrast, GDD patients have normal stature, sclera, and hearing (Otaify et al., 2018). In addition, in recent years it has shown that the genetic characteristics distinguish GDD as a distinct entity.

GDD has been first described by Akasaka et al. (1969) in a Japanese pedigree, but to date, other families of Italian, American, Asian Indians, Caucasian, and Chinese, as well as sporadic case reports have been documented (Duong et al., 2016; Marconi et al., 2013; Rolvien et al., 2017, Sandal et al., 2021; Tsunami et al., 2003, 2004). Tsunami et al. (2003, 2004) showed that mutations in *anoctamin 5 (ANO5*; OMIM #608662*)* gene are responsible for the molecular cause of GDD, which is a protein‐coding gene within the GDD critical region on 11p15.1‐p14.3. Moreover, the autosomal recessive muscular dystrophies, including limb‐girdle muscular dystrophy type 2 L and Miyoshi myopathy type 3 have also shown to be associated with *ANO5* mutations (Bolduc et al., 2010; Mahjneh et al., 2010). *ANO5* plays the important role in various physiological processes, such as ion transport, phospholipid scrambling, and regulation of some ion channels (Xu et al., 2015).

Here we report a large Iranian family with autosomal dominant GDD caused by a newly identified missense variant, NM_213599.3:c.1078T>C (p.(Cys360Arg), in the *ANO5*, using a next generation sequencing (NGS) approach. To the best of our knowledge, NM_213599.3:c.1078T>C is a novel pathogenic variant associated with GDD.

## 2 MATERIALS AND METHODS

**FIGURE 1:**
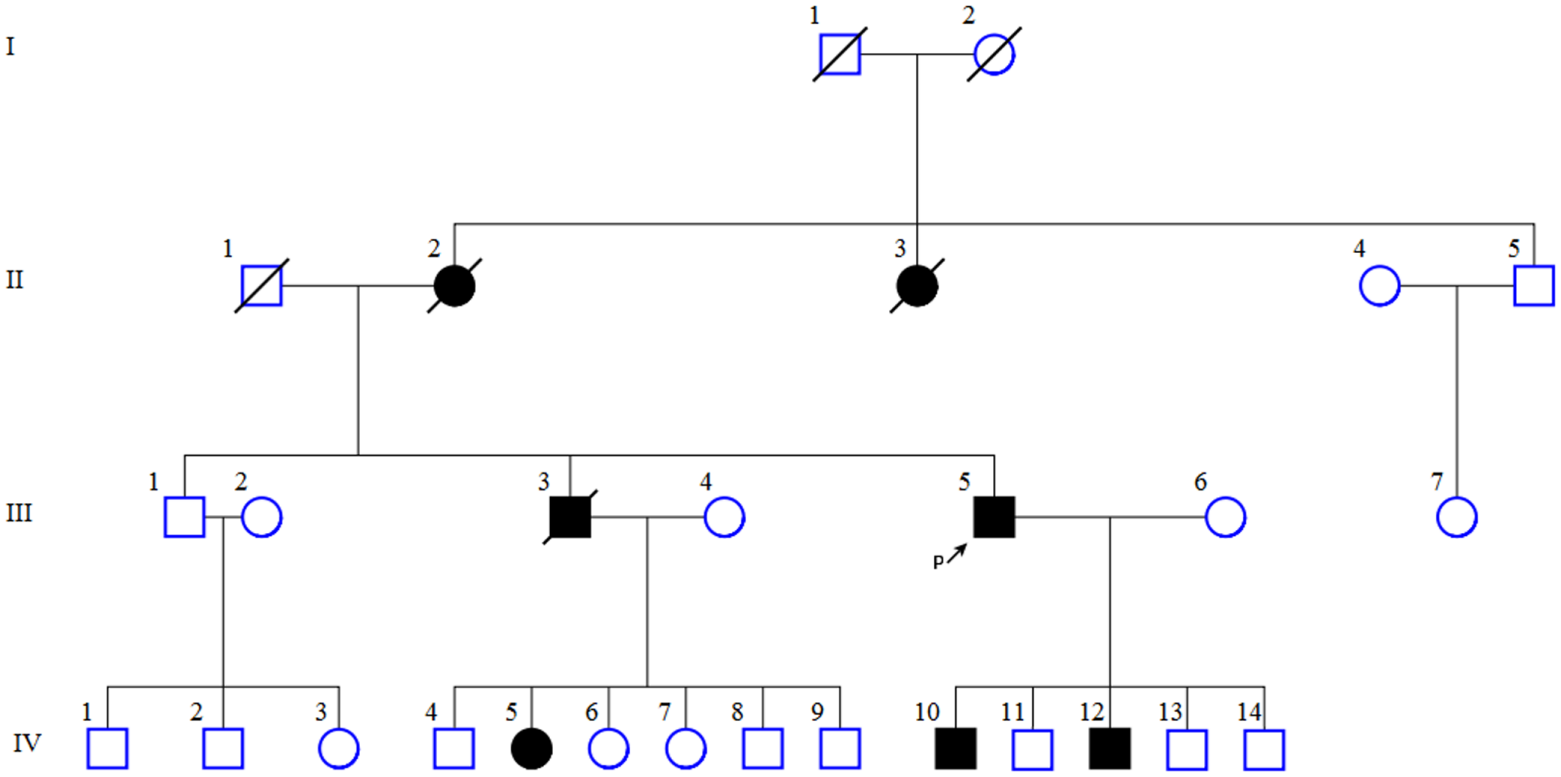
Pedigree of the four‐generation family with autosomal dominant gnathodiaphyseal dysplasia (GDD). Black symbols represent the affected individuals and open symbols represent normal individuals. The black arrow (P↑) represents the proband III‐5. DNA samples of individual in this pedigree were analyzed by sanger sequencing. DNA samples of I‐1, I‐2, II‐1, II‐2, II‐3and III‐3 were not available.

All procedures in this study followed the tenets of the Declaration of Helsinki. Also, this study was approved by ethical committee of Shahid Beheshti University of Medical Science. Informed written consent to publish the clinical data and the photographs was obtained from participants and for the patients under the age 18 years‐old, from their parents. Family histories, detailed clinical information and blood samples were collected from all individuals in the pedigree (Figure 1).

The DNA was extracted from blood samples using the standard salting‐out method (Miller et al., 1988) and stored at −20°C until use. Exome sequencing (ES) was performed for the proband III‐5 (Figure 1). Briefly, the library was prepared using the Agilent SureSelectV6‐post kit (Agilent Technologies, Ltd), according to manufacturer's instructions. The products had an average fragment size of 250 bp. The library was sequenced on an Illumina Hiseq 4000 machine (Illumina, lnc.). The sequencing data were aligned to the human reference genome builds b37 using BWA (Genomes Project et al., 2010; Li et al., 2010). BAM file was prepared for variant calling using SAMtools and Picard tools (Li et al., 2009). Variant calling was performed using GATK and SAMtools, followed by variant annotation using SNPEff (McKenna et al., 2010; Cingolani et al., 2012). Rare variants with a minor allele frequency (MAF) <0.1%, including nonsense, synonymous, nonsynonymous, splice site, insertion, and deletions were selected for interpretation.

### 2.1 Mutation assessment and structural modeling

Bioinformatics tools were applied to test the pathogenicity of the variant (NM_213599.3:c.1078T>C), including MutationTaster, SIFT, EIGEN, EIGEN PC, MutPred, REVEL, BayesDel addAF, BayesDel noAF, FATHMM‐MKL, FATHMM‐XF, MetaLR, MetaSVM, PROVEAN, and SIFT4G (https://varsome.com/). By using the Phyre2 (Kelley et al., 2015), we analyzed the effects of the detected variant on the protein structure and function. Furthermore, the homology modeling visualized by PyMol (V 1.3).

### 2.2 Polymerase chain reaction (PCR) and sanger sequencing

We amplified the exon 11 and flanking intronic regions of the *ANO5* in DNA samples obtained from all individuals in the pedigree (excluding I‐1, I‐2,II‐1, II‐2, II‐3 and III‐3) with specific primers, designed by Gene Runner software (version 3.05). The primer sequences are shown in Table 1. Sanger sequencing was performed to confirm the variant identified by ES.

**TABLE 1:** Primer sequences for *ANO5* gene amplification. This primer set amplify the exon 11 and flanking intronic regions of *ANO5* gene

Primer name	Primer sequence (5′–3′)	Lengh (bp)	Ta	Product size (bp)
ANO5‐11F	GGCTCTGAAAACTCTACTCCTC	22	59	605
ANO5‐11R	CCACTAATATGCACTCCATCAG	22		

*Note*: GenBank reference sequence: NM_213599.3.

Abbreviations: bp, base pair; F, forward; R, reverse; Ta, annealing temperature.

### 2.3 Segregation analysis

The presence of candidate variant was traced within all available family members using PCR and Sanger sequencing.

## 3 RESULTS

### 3.1 Clinical description and samples

**FIGURE 2:**
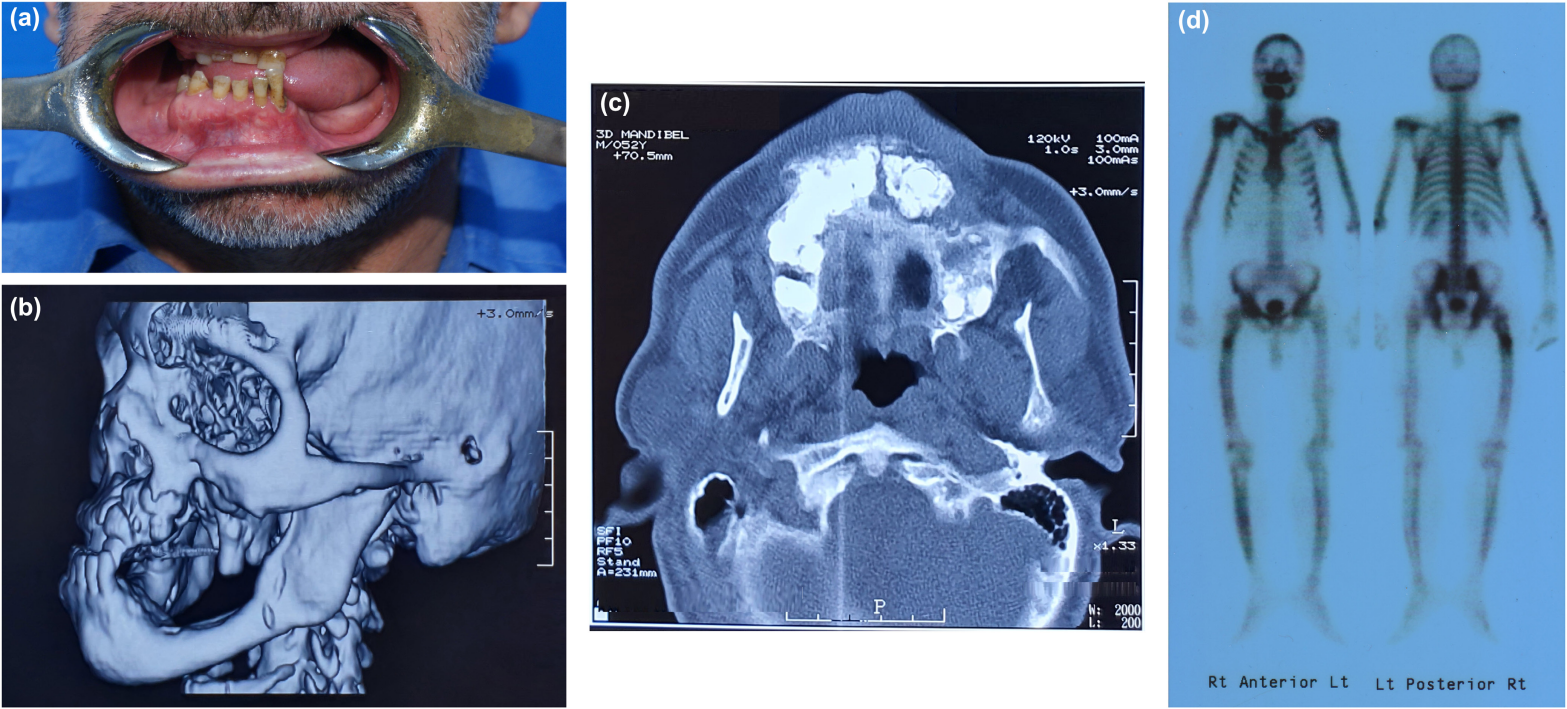
(a) Clinical view of severe mandibular resorbtion of the proband. (b) Magnitic resonance imaging (AP view) and (c) computed tomography scan (axial view) shown at age of 51 years of proband. They revealed patient's mandible cortex destruction with lytic sclorotic and suspected fat fracture in right alveolar ridge. (d) PET scan findings: TC99m whole body bone scintigraphy is shown at age of 49 years of proband. Foci with abnormal intense tracer concentration are visualized in maxilla and mandible considering cementoblastoma. In addition, increased tracer localization is also seen in other osseous structure, with bowing of right femur, tibiae, likely due to the osteoporosis. A zone of mild to moderate increase tracer uptake in the upper third of right femur, is likely inflammatory infectious process.

A 48‐years old man of Iranian descent (Figure 1‐III‐5 and Table 2), who presented with a history of a slowly enlarging chin and chronic pus discharge from the left side of the posterior maxilla to the oral and maxillofacial department in Taleghani hospital. His symptom was initially begun at the age 22 years, when a mandibular fracture occurred during the extraction of the tooth 19. Imaging results including MRI, CT and PET scan revealed mandible, maxilla mixed lytic and sclorotic appearance and long bone deformity (Figure 2a–d). The fracture was reduced and stabilized with a wire, but osteomyelitis developed, and continual pus drainage was observed 9 months post‐surgery. The tissues from the intervention were submitted for histological analysis, which revealed a lesion to be cementum‐like masses, rounded margins surrounded by active fibrosis. Multiple, widespread, mixed radiodensity lesions were predominant in both jaws. Limb radiographs disclosed osteopenic bone and excess callus in the fracture area. The patient was treated by partial maxillectomy and oral rehabilitation was accomplished with the partial removable denture. His past medical history was significant for multiple prior bone fractures, including a left proximal femur and humorous bones fractures due to minor trauma at the age of 42 years.

**TABLE 2:** Clinical features of GDD patients

Patirnt	Sex	Age of onset	Jaw lesions	Bone fractures			
				Jaw	Hand	Foot	Hip
III3	Male	No data	Yes	Yes	Yes	Yes	Yes
III5	Male	22 years	Yes	Yes	Yes (4 times)	Yes	Yes
IV5	Female	16 years	Yes	Yes	Yes (1 time)	no	no
IV10	Male	9 years	Yes	yes (at 36 years)	Yes (3 times)	Yes (3 times)	Yes (1 time)
IV12	Male	32 years	Yes	Yes	No	No	No

Family history was significant for a mandibular tumor that developed in his brother at the age of 51 (III‐3).His symptoms that have been begun at the age of 25 years consisted of an expansion of the bony cortical palate in which was first noticed on the anterior ridge of the mandible, when chronic osteomyelitis had developed in the area after tooth extraction. Alveoloplasty with curettage of the necrotic bone was carried out, followed by prosthodontics rehabilitation. The specimen biopsy derived from this region, revealed histological pattern similar to those found in his brother. Radiographs of the jaws shown large, well‐defined, lobular, mixed radiolucent/radiopaque masses surrounded by a narrow radiolucent zone. Likewise, he had a history of non‐trumatic multiple long bones fractures between the age of 35 and 45 years.

The two sons of the proband (III‐5) were also clinically and radiographically investigated. His 25‐years old son (IV‐10) had a history of femur bone fractures as well as extensive mixed radiodensity lesions spread in all four quadrants of the jaws in his radiographs. His younger 20 years old son (IV‐12) has no history of long bone fractures up to now. However, he had radiolucent lesions at the apices of the maxillary teeth. Conversely, no bone lesions were observed in his radiological examinations at the age of 17 years. Moreover, patient II‐2 and II‐3 had the same condition (Table 2).

### 3.2 Mutation identification

**FIGURE 3:**
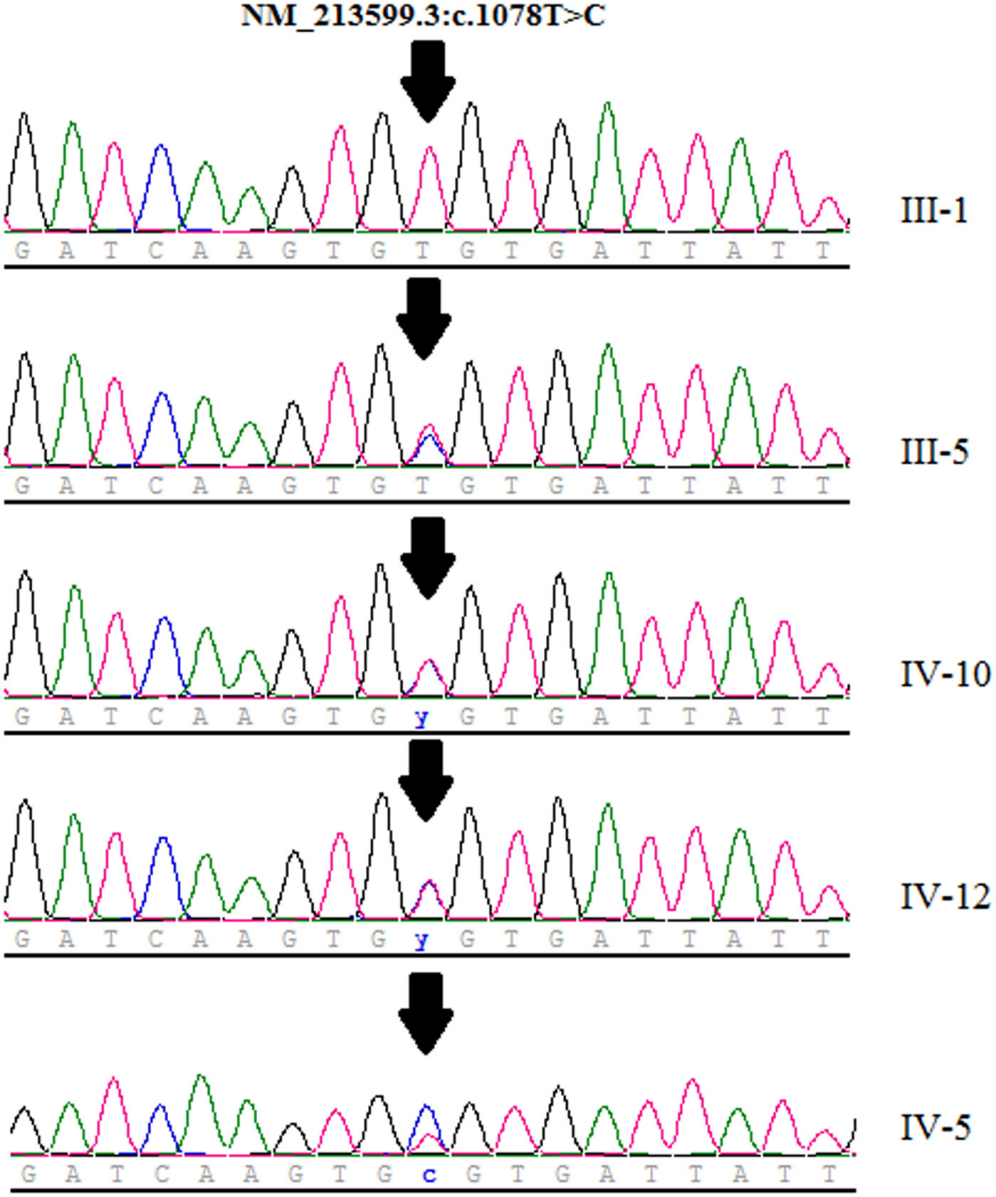
Electropherogram for exon 11 of *ANO5* gene (NM_213599.3) for the proband (IV3), and other patients (IV‐5, IV‐10, and IV‐12). After designing specific primers, PCR was done. PCR products were used for sanger sequencing. The arrow symbol indicates the heterozygous mutation c.1078T>C in the *ANO5*.

Exome sequencing of the proband (III‐5) was performed and revealed a novel heterozygous pathogenic variant, NM_213599.3:c.1078T>C (p.Cys360Arg) in the *ANO5* gene. This variant is located in the putative extracellular loops of human ANO5, and is conserved in humans, teleost, and insect species. Sanger sequencing results confirmed this variant in proband (Figure 3) and in all affected individuals (IV‐5, IV‐10 and IV‐12) in the pedigree, which segregated with the disease within the family. This variant led to the replacement of cysteine with arginine amino acid. This variant was classified as pathogenic, based on the American College of Medical Genetics and Genomics interpretation guideline (ACMG; Richards et al., 2015) which is consisted of presence of the dense hot‐spot region, conserved amino acid position, pathogenic computational verdict, and ClinVar classification as well as literature review.

### 3.3 In silico analysis

**FIGURE 4:**
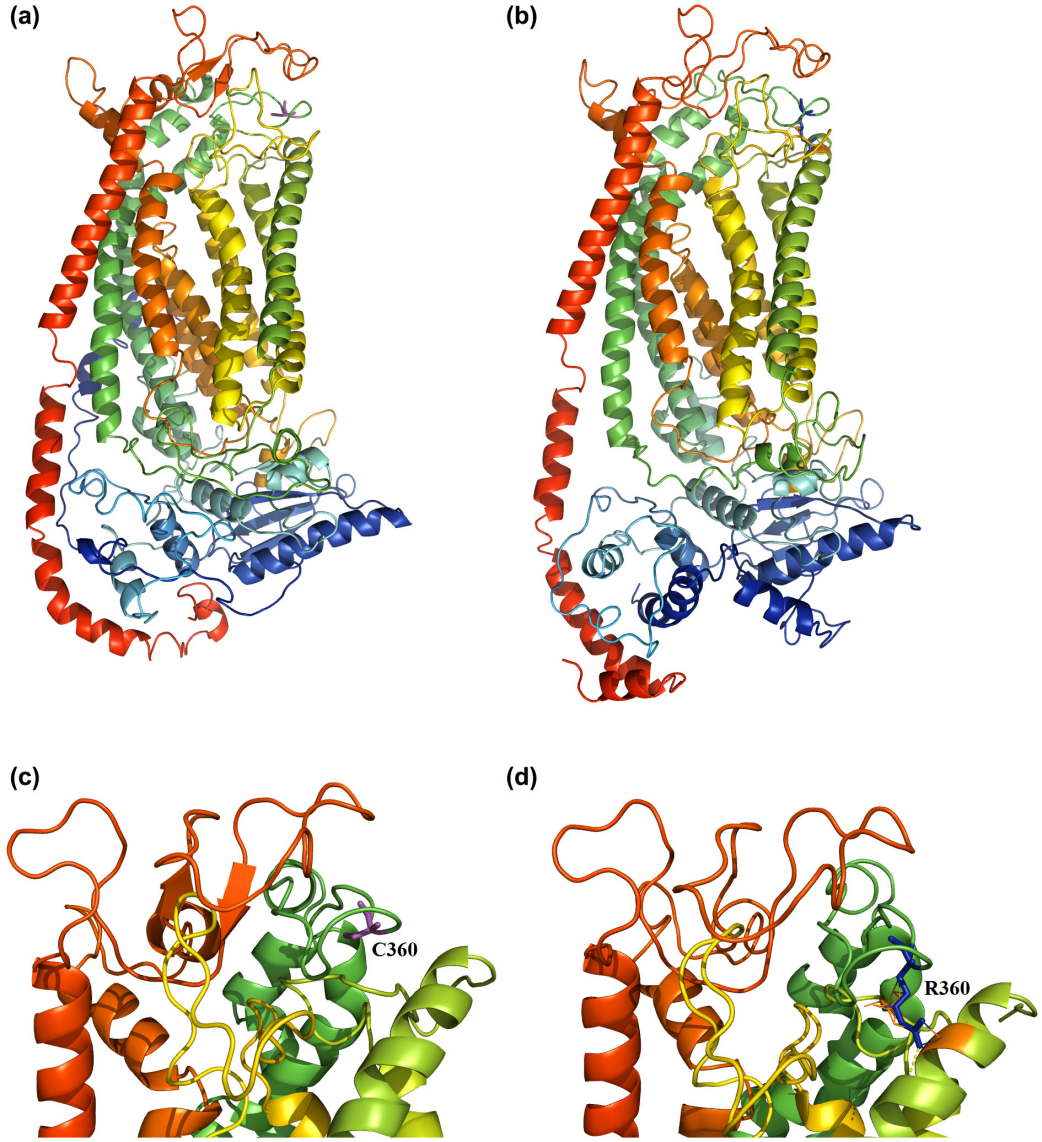
Predicted structure of wild‐type and mutant human ANO5 protein. Protein modeling analysis showed that the mutations detected in ANO5 protein make structural differences compared to wild type protein. (a) Wild‐type ANO5, and B) C360R‐mutated ANO5. The location of cysteine (in magenta) and mutant arginine (in blue) at position 360 are shown. Figures (c) and (d) illustrate a close view of wild‐type and mutated residues of extracellular domain, respectively. 3‐dimensional structures showed that substitution of cysteine by arginine at position 360 changes the structure of the extracellular loop as well as domains that are in close proximity of this amino acid. This variant might damage the correct protein folding and dimerization of monomers leading to loss of function of ANO5 protein.

**FIGURE 5:**
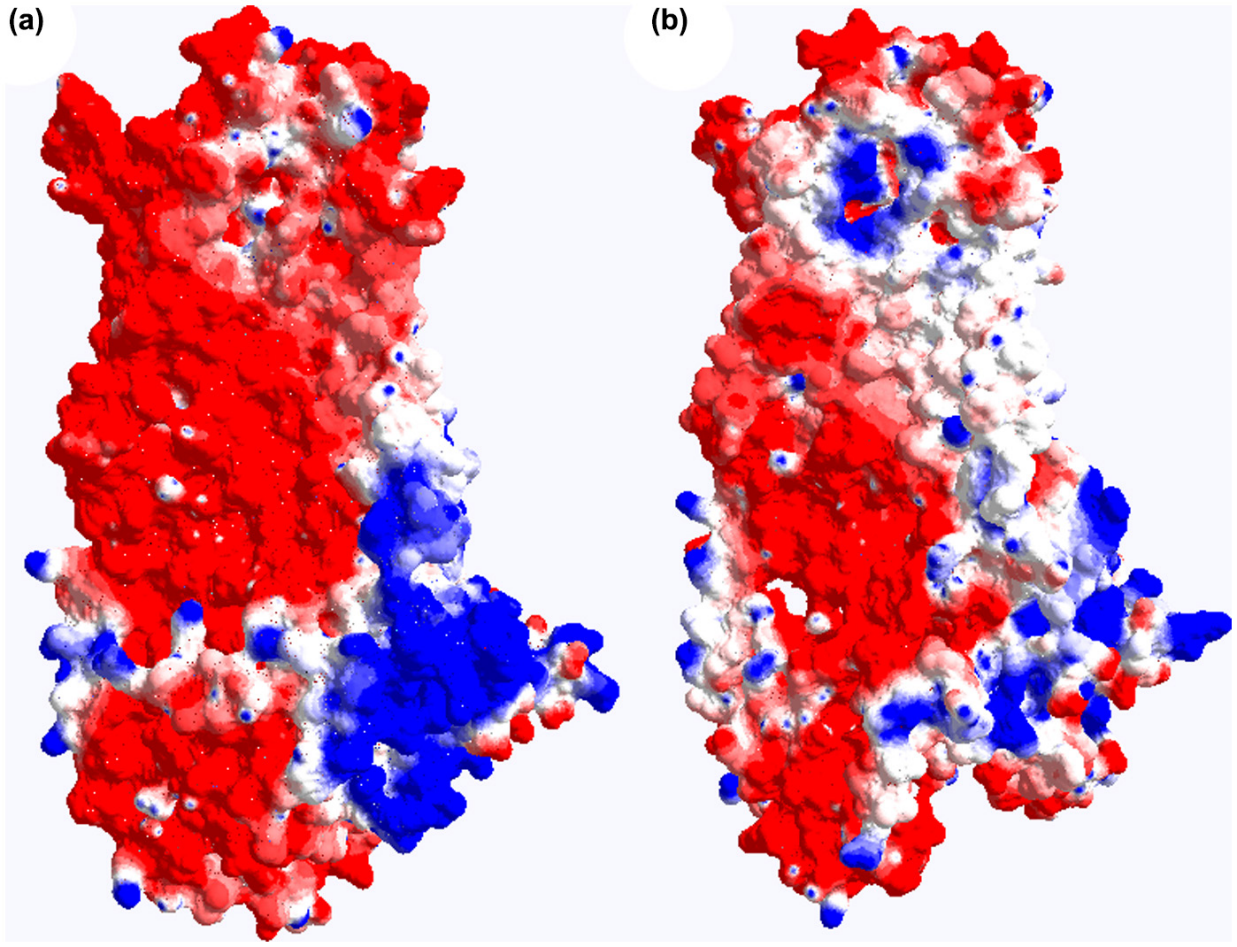
Surface electrostatic images of ANO5 protein. (a) Wild‐type ANO5 and (b) Cys360Arg ‐mutated ANO5. The Cys360Arg mutation led to alteration in the electrostatic characteristics of the extracellular region.

In silico analysis predicted the variant as a pathogenic (EIGEN, EIGEN PC, MutPred and REVEL), disease causing (MutationTaster), and damaging (BayesDel addAF, BayesDel noAF, FATHMM‐MKL, FATHMM‐XF, MetaLR, MetaSVM, PROVEAN, SIFT, and SIFT4G) variant. Homology modeling of ANO5 was performed using Phyre2 (V 2.0) and visualized by PyMol (V 1.3). Two templates, c6p46A and c6qpbB were selected to model proteins based on heuristics to maximize confidence, percentage identity, and alignment coverage. Finally, 155 residues (83% of residues) were modeled (at >90% confidence) by using Phyre2. ANO5 homology modeling displayed the structural alteration compared to intact protein (Figures 4 and 5).

## 4 DISCUSSION

Although the molecular mechanism associated with GDD is not fully understood, however, the role of mutations in the *ANO5* gene is clear in the disease pathogenesis. ANO5 protein is a member of the anoctamin family of transmembrane proteins, which is a calcium‐activated chloride channel sharing a common structural feature of eight‐transmembrane domains (Jin et al., 2017).

As shown in Table 3, several studies have reported that *ANO5* mutations are associated with GDD. Moreover, numerous types of identified variants in *ANO5* are available at HGMD®. Zeng et al. (2019) have reported a novel mutation c.1067G>T (p.C356F) in *ANO5* which is the causes of GDD. This mutation has been specified as a deleterious variant by bioinformatics analyses and structural modeling. Marechal et al. (2019) reported a family with a novel *ANO5* mutation: c.1790G>T (p.Arg597Ile). Jin et al. (2017) identified a *ANO5* gene mutation, c.1079G>A (p.Cys360Tyr), in a Chinese family with jaw infection and cementoma who suffered from purulent osteomyelitis‐like symptoms including purulent discharge from gum, tooth mobility, loss of teethes and insufficient healing after dental extraction without multiple fractures. The similar symptoms of the patient mentioned by Jin et al. (2017) were seen in our study, although our patients exhibited bone fractures (patient III‐5, V‐5, and V‐10) in earlier age.

**TABLE 3:** *ANO5* mutations associated with gnathodiaphyseal dysplasia

Mutation:RNA level	Mutation: protein level	Mutation type	ACMG classification	Reference
c.1067G>T	p.Cys356Tyr	Missense	Likely pathogenic	Zeng et al. (2019)
c.1067G>A	p.Cys356Tyr	Missense	Likely pathogenic	Jin et al. (2017)
c.1079G>A	p.Cys360Tyr	Missense	Likely pathogenic	
c.1553G>A	p.Gly518Glu	Missense	Likely pathogenic	
c.643A>G	p.Arg215Gly	Missense	Uncertain significance	
c.1067G>A	p.Cys356Tyr	Missense	Likely pathogenic	Andreeva et al. (2016)
c.1499C>T	p.Ser500Phe	Missense	Uncertain significance	Rolvien et al. (2017)
c.1790G>T	p.Arg597Ile	Missense	Uncertain significance	Marechal et al. (2019)
c.1066T>C	p.Cys356Arg	Missense	Likely pathogenic	Tsutsumi et al. (2004)
c.1066T>G	p.Cys356Gly	Missense	Likely pathogenic	
c.1067G>A	p.Cys356Tyr	Missense	Likely pathogenic	Duong et al. (2016)
c.1553G>A	p.Gly518Glu	Missense	Likely pathogenic	Otaify et al. (2018)
c.1538C>T	p.Thr513Ile	Missense	Uncertain significance	Marconi et al. (2013)
c.1067G>A	p.Cys356Tyr	Missense	Likely pathogenic	Vengoechea and Carpenter (2015)
c.1078T>C	p.Cys360Arg	Missense	Pathogenic	Current study

The c.1078T>C and c.1079G>A cause change of Cysteine to Argenine and Cysteine to Tyrosine amino acid in the codon p.360 in the present study and previously reported by Jin et al. (2017), respectively. It is possible that changing cysteine to Arginine may result in more incorrect folding and function of ANO5 and penetrance of the disease in earlier age.

In this study, molecular analysis of the proband using ES, discovered a novel nucleotide alteration, leading to an amino acid exchange of cysteine to arginine at position 360, in an Iranian family with autosomal dominant GDD. This variant was fully segregated with the disease phenotype in all the affected family members. The c.1078T>C was predicted as pathogenic based on the ACMG (Richards et al., 2015). Evolutionary conservation analysis indicated that the p.(Cys360Arg) variant resulted in a conserved codon exchange (Tsutsumi et al., 2004).

As depicted in Figure 4, mutated model of ANO5 protein (p.C360R) showed a different structure of the extracellular loop as well as domains that are in close proximity of this amino acid in comparison to the structure of the wild‐type protein. Furthermore, this variant led to alteration in the electrostatic characteristics of the extracellular region (Figure 5). Jin et al. (2017), by performing functional studies, showed that mutation at this location (p.C360Y) reduces gene expression. Moreover, they showed cysteine residues in the putative extracellular loops may be important for the folding of the ANO5 protein by participating in intrachain bonds. In agreement with our results, these concordant pieces of evidence and the phenotype of the patients (were completely explained in the Materials and Methods section) suggested that *ANO5*: NM_213599.3: c.1078T>C pathogenic variant is possible cause of patients' phenotype in the studied pedigree.

Li et al. (2022) by generating the first knock‐in mouse model for GDD, showed that *Ano5* knock‐in mice (*Ano5* ^*KI/KI*^) replicated GDD‐like skeletal features. However, Xu et al. (2015) by generating the first *Ano5*‐knock‐out mice showed that genetic ablation that does not cause over pathology in its skeletal and cardiac muscles. GDD patients have shown variable age of onset and expression of the disease phenotype, even within the same family whose affected members all shared the same mutation, which may result from mutations in potential modifier genes, including *COL5A1* (Andreeva et al., 2016; Jin et al., 2017).

ES is quite beneficial molecular approach when the clinical signs/symptoms and genetic variant of the patient are not sufficient for accurate diagnosis.

In summary, we report a novel possibly pathogenic variant NM_213599.3:c.1078T>C (p.C360R) in *ANO5* gene in an Iranian family with GDD. Although the codon position identified in our study was also found in Jin et al. (2017) report, however, differences in the type of amino acid p.(Cys360Arg) produced in our patient may cause symptoms to begin at an earlier age and differences in the type of symptoms. However, further studies are needed to confirm this conclusion. Our result showed ES may accelerate the discovery of pathogenic variant and diagnosis the disease precisely. Moreover, early diagnosis is critical, because the patient may take benefit from early intervention. Furthermore, genetic counseling of asymptomatic individuals and their relatives should be offered for precautionary measures.

## AUTHOR CONTRIBUTIONS

Vahid Reza Yassaee and Arash Khojasteh performed the clinical evaluation of patients and interpreted the data. Farzad Hashemi‐Gorji collected data, designed and performed molecular analyses, interpreted the molecular data, performed protein modeling and wrote the manuscript. Hossein Sadeghi, Hannaneh Safiaghdam, and Reza Mirfakhraie collected data and wrote the manuscript. All authors reviewed and approved the manuscript.

## CONFLICT OF INTEREST

The authors declare that they have no conflict of interest.

## Data Availability

The data that support the findings of this study are available from the corresponding author upon reasonable request.
